# Supplementary material for: Family-GWAS reveals effects of environment and mating on genetic associations
Source: medRxiv. 2025 Oct 14:2024.10.01.24314703. Preprint. [Version 2] doi: 10.1101/2024.10.01.24314703 (PMC12633112; doi:10.1101/2024.10.01.24314703)
Supplement: Supplement 3 [file media-3.pdf]

Analysis plan for ‘Meta-analysis of Mendelian Imputation based Genome-Wide Association Studies’

Overview

We are conducting a meta-analysis of family-based genome wide association studies aided by Mendelian Imputation of missing parental genotypes. The underlying model for the effect of a SNP on a phenotype is:

$$E\left[Y_{ij} \middle| g_{ij}, g_{p(i)}, g_{m(i)}\right] = \delta g_{ij} + \beta_p g_{p(i)} + \beta_m g_{m(i)},$$

where Y_{ij} is the phenotype of sibling j in family i, g_{ij} is the genotype of that individual at the SNP, $g_{p(i)}$ is the genotype of the father, and $g_{m(i)}$ is the genotype of the mother; δ is the direct effect of the SNP, β_p is the ‘paternal effect’ of the SNP, and β_m is the ‘maternal effect’ of the SNP.

When parental genotypes are unobserved, they are imputed from the observed genotypes in the family according to the inferred inheritance pattern (Mendelian Imputation), where identity-by-descent sharing information is leveraged as appropriate: see https://www.biorxiv.org/content/10.1101/2020.07.02.185199v1.abstract.

For cohorts where no parental genotypes are observed, the imputed maternal and paternal genotypes are the same, so we use the imputed sum of maternal and paternal genotypes and estimate δ and $(\beta_p + \beta_m)/2$. Despite the fact that some cohorts cannot directly estimate β_p and β_m separately, we will use a form of multivariate meta-analysis to produce meta-analysis estimate of δ, β_p, β_m for each SNP. From these meta-analysis estimates of the underling parameter vector, we will be able to produce meta-analysis estimate of linear transformations of this parameter vector, including average parental effects, $(\beta_p + \beta_m)/2$, and ‘population effects’, $\delta + (\beta_p + \beta_m)/2$, as estimated by standard GWAS of unrelated individuals.

Phenotypes

Phenotype	Age restriction	Coding/notes
ADHD	None	Clinical diagnosis or self-report; binary coding
Age at first birth	NA	NA
age at menarche	NA	NA
asthma	None	ICD10, self-report (of diagnosis or symptoms) or clinical diagnosis; binary coding
Eczema (atopic dermatitis)	None	ICD10, self-report (of diagnosis or symptoms) or clinical diagnosis; binary coding
BMI	18+	None
HDL Cholesterol	18+	None
Non-HDL cholesterol	18+	Total cholesterol-HDL cholesterol
Blood pressure	18+	Both diastolic and systolic
ever cannabis	18+	Self-report; Binary coding
cigarettes per day	18+	both former and current smokers; those with no data set to missing
Drinks-per-week	18+	Self-report; quantitative, midpoint of range used if response is binned
depression	18+	self-report (of diagnosis or symptoms) or clinical diagnosis; binary coding; exclusions for individuals identified as suffering from other mental illnesses
Depressive symptoms	18+	Use as quantitative scale
educational attainment	30+	Map to ISCED-1995 categories and convert to US years of schooling equivalent; contact for help with this
Cognitive ability/IQ	None	Use most complete measure of general cognitive ability
ever smoker	18+	Binary coding
extraversion	18+	scores based on answers to personality trait tests; quantitative
FEV1	18+	Forced expiratory volume in 1 second (lung health measure)
hayfever	None	ICD10, self-report (of diagnosis or symptoms) or clinical diagnosis; binary coding
height	18+	None
Household income	18+	self-report: if binned variable analyze by treating the midpoints of categories of income as a continuous variable; log scale
Hourly income	18+	Imputed from occupation using survey data; transformed to log scale; quantitative
migraine	18+	self-report or clinical diagnosis or diagnosis from questionnaire responses; binary coding
morning person	18+	Self-report; Binary coding
nearsightedness	None	questionnaire responses or self-report (of diagnosis or symptoms); binary coding

number ever born	45+ for men; 55+ for women	None
neuroticism	18+	scores based on answers to personality trait tests; quantitative
self-rated health	18+	Use as quantitative scale from 1 (worst self-rated health) to N (best self-rated health), where N is the number of possible ranked answers to survey question
subjective well-being	18+	Use as quantitative scale from 1 (worst subjective well-being) to N (best subjective well-being), where N is the number of possible ranked answers to survey question; combine scores from questions for 'positive affect' and 'life satisfaction' if appropriate

Cohort Level Analyses

1. Filter variants for MAF>1% and INFO>0.99, or equivalent imputation information metric, and convert to .bed format. (If phased haplotypes are available, do the same and put in phased .bgen format.)
2. Run KING with –related –degree 1 options to infer first degree relatives
3. Run KING with –ibdsegs –degree 1 options to infer IBD segments between first-degree relatives
4. Download and install SNIPar: https://github.com/AlexTISYoung/SNIPar
5. Run tests (python setup.py pytest) to ensure correct compilation and installation
6. Impute each chromosome separately using the impute_runnner.py script: see the tutorial for examples https://github.com/AlexTISYoung/SNIPar/blob/master/docs/tutorial.rst. If memory is an issue, use the –chunks argument to read the SNPs in chunks to reduce maximum memory usage. The script takes the IBD segments and relations inferred by KING along with a plain text file giving the age and sex of each individual (columns FID, IID, age, sex), with sex coded as 'M' for male or 'F' for female. These are used to construct a pedigree and to perform the imputation based on the pedigree and the IBD segments. Alternatively, a user-input pedigree can be provided with columns FID, IID, FATHER_ID, MOTHER_ID
7. Run fGWAS.py script for each chromosome for each phenotype. Phenotypes should be adjusted for age at measurement (and year-of-birth if different), sex, age^2, age^3, age*sex, age^2*sex, age^3*sex and the top 20 principal components. This can be done before the phenotype is input to the fGWAS.py, or the covariates can be provided to the fGWAS.py script. The script takes the observed genotypes, the imputed genotypes, and the phenotype file as minimal inputs. This will output HDF5 and gzipped text summary statistics.
8. Send text and HDF5 summary statistics files to alextisyoung@gmail.com. Please also send any other imputation/genotyping quality metrics on the SNPs, such as imputation

INFO, call-rate, and Hardy-Weinberg Equilibrium P-value. Please also send the allsegs output from the KING IBD inference that contains the intervals where IBD is assessed.

Meta-analysis and downstream analyses

1. We will estimate the variance due to direct and population effects within each cohort, as well as their correlation. We will check the correlation between the population effects and publicly available summary statistics as a form of quality control.
2. We will use METAL and multivariate meta-analysis to obtain meta-analysis estimates of direct, paternal, and maternal effects at each SNP for each phenotype.
3. We will construct polygenic indexes based on direct effects only for each phenotype and test their predictive ability in the population and within-families in left-out prediction cohorts.
4. We will estimate genetic correlations between phenotypes using direct-effect summary statistics.
5. For each phenotype, we will estimate genetic correlations between direct and average parental effects and between direct and population effects.
